# Supplementary material for: Effector Profiles of Endophytic Fusarium Associated with Asymptomatic Banana (Musa sp.) Hosts
Source: Int J Mol Sci. 2021 Mar 2;22(5):2508. doi: 10.3390/ijms22052508 (PMC7975973; doi:10.3390/ijms22052508)
Supplement: Supplementary file 1 [file ijms-22-02508-s001.zip › IJMS_Czislowski_131220_Supplementary/Supplementary Figures.docx]

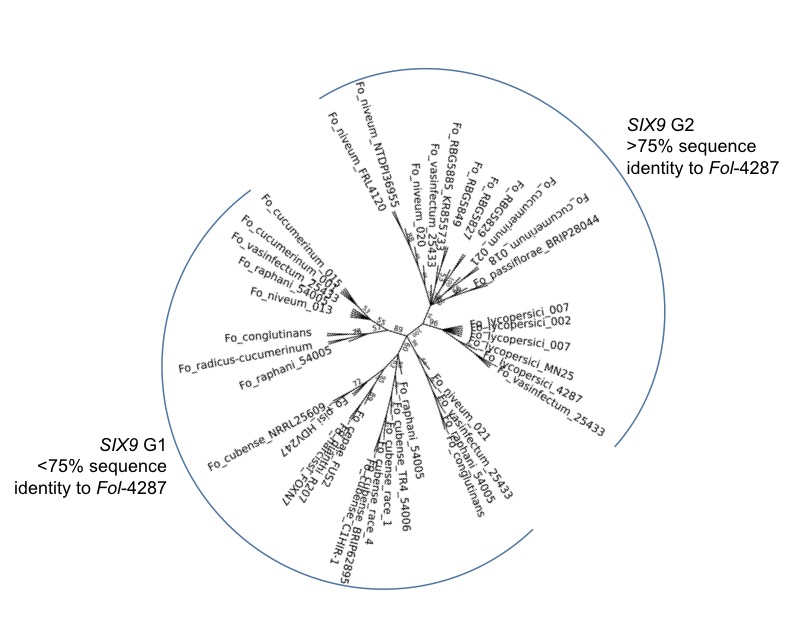


**Figure 1** Neighbour-joining tree of *SIX9* homologues identified using a BLAST from the nucleotide database and genomes of *F. oxysporum* available on the whole genome data base (NCBI). Branch labels indicate support values derived from 100 bootstraps. Homologues of *SIX9* that showed <75% sequence identity to the reference sequence of *Fol-007* (indicated with red circle, GenBank accession KC701447.1; Schmidt et al., 2013) were designated *SIX9-*group 1. Homologues that showed >75% sequence identity to the *SIX9* reference sequence from *Fol-007* were designated *SIX9*-group 2.

**Supplementary Figure 2** Gene trees of *SIX* genes identified and sequenced in this study. Trees were inferred using Bayesian analysis. Internal node support is indicated as Bayesian probabilities.

*SIX4*

z


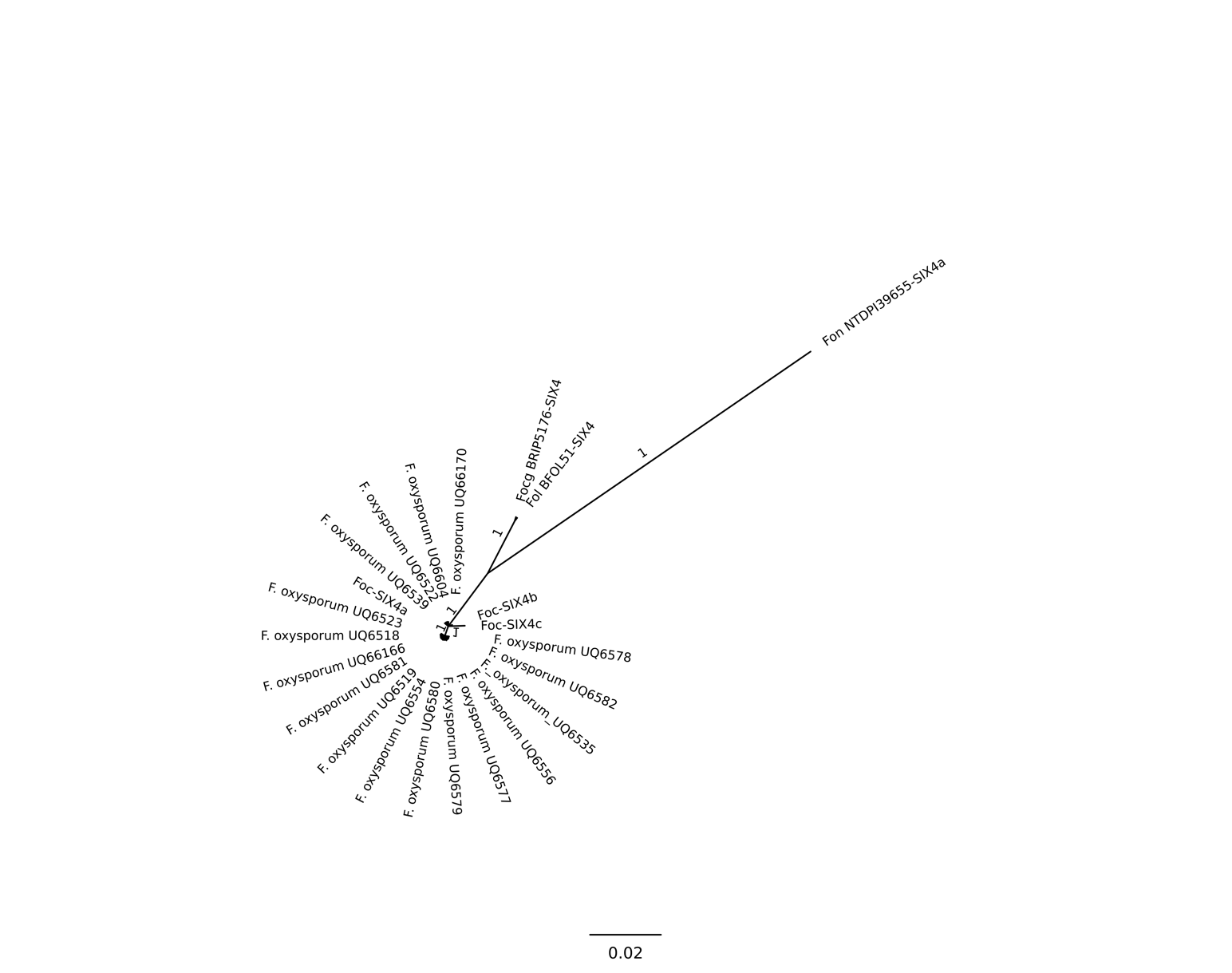


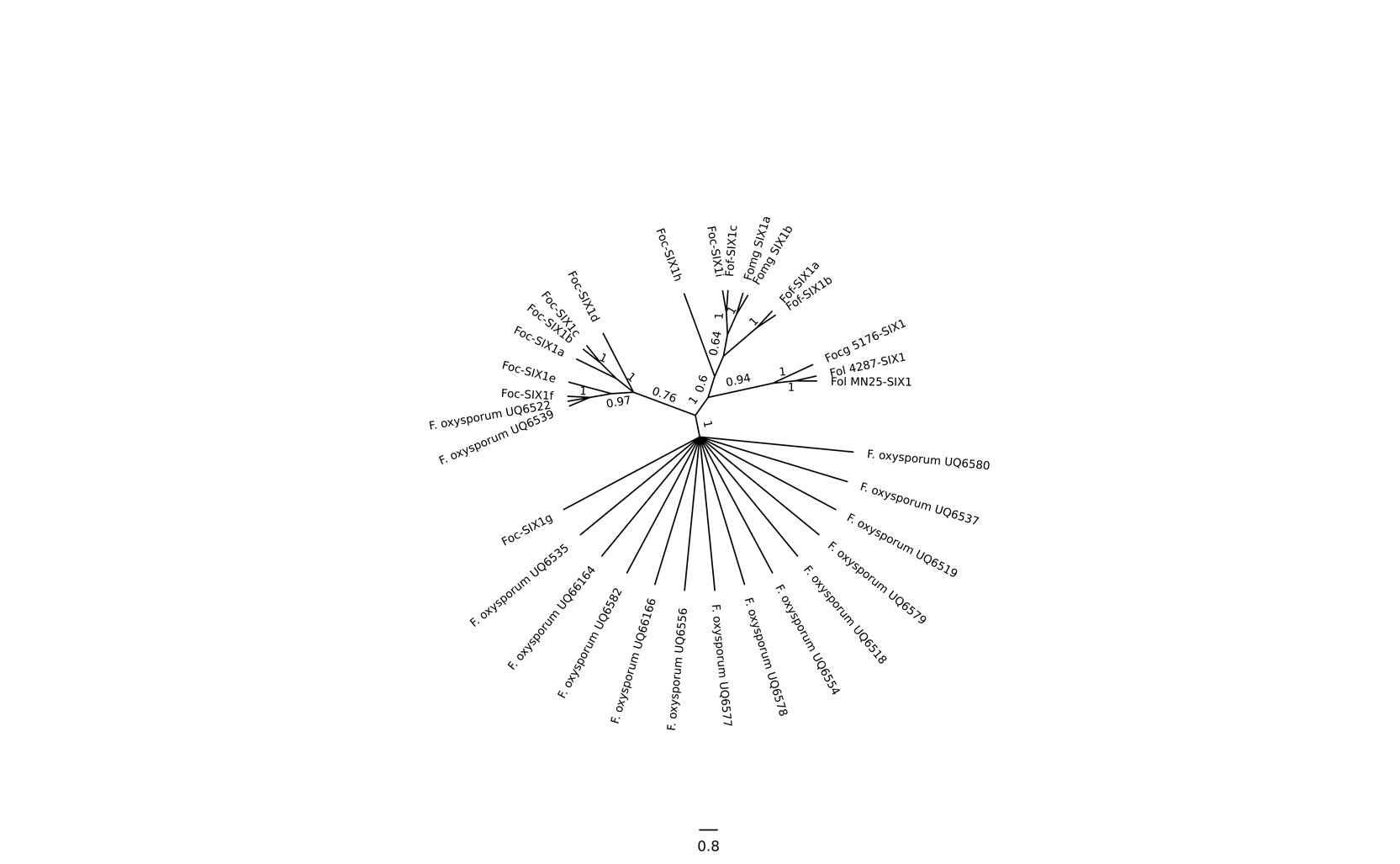

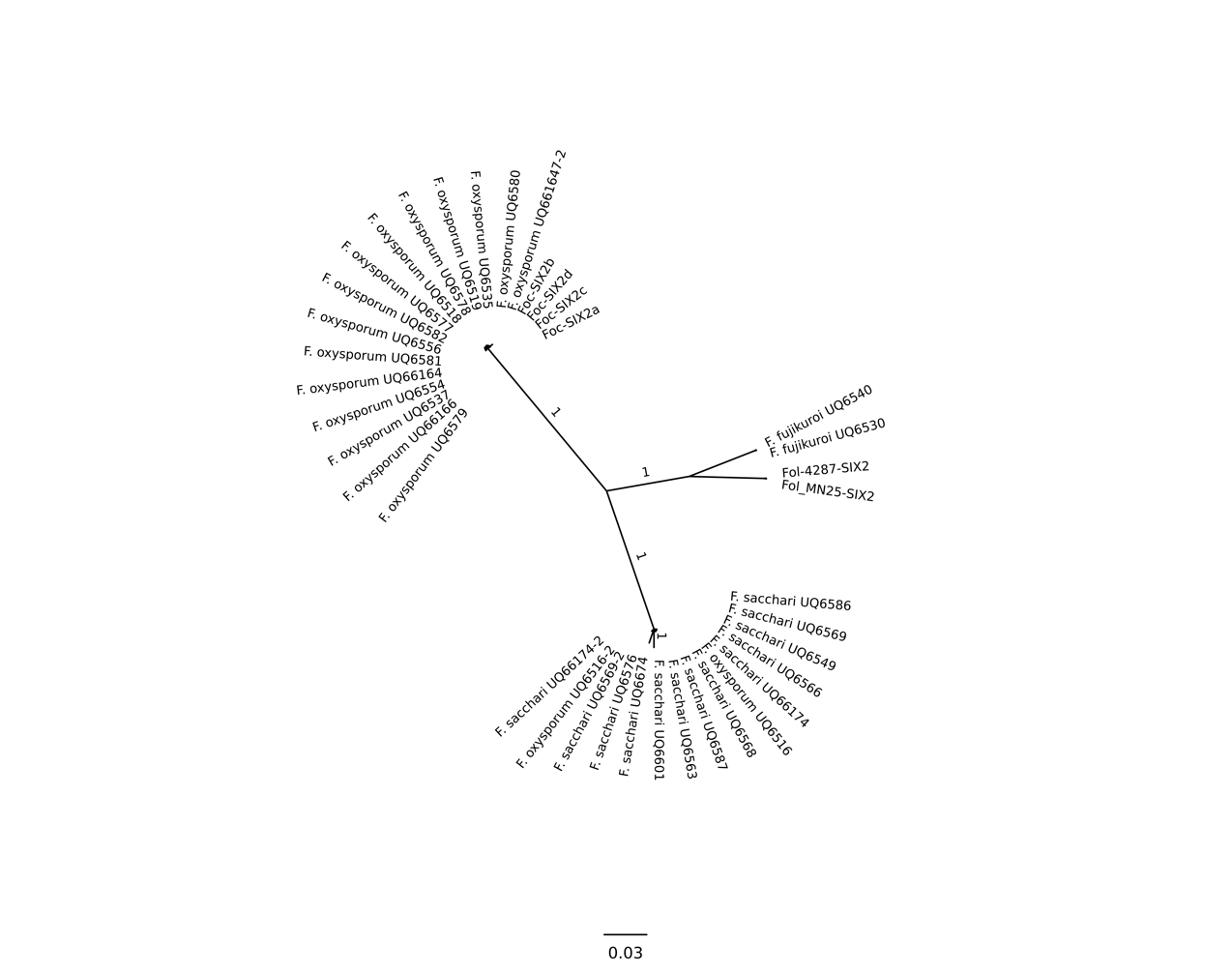


*SIX2*

*SIX1*

Zz

z

**Supplementary Figure 2** continued…


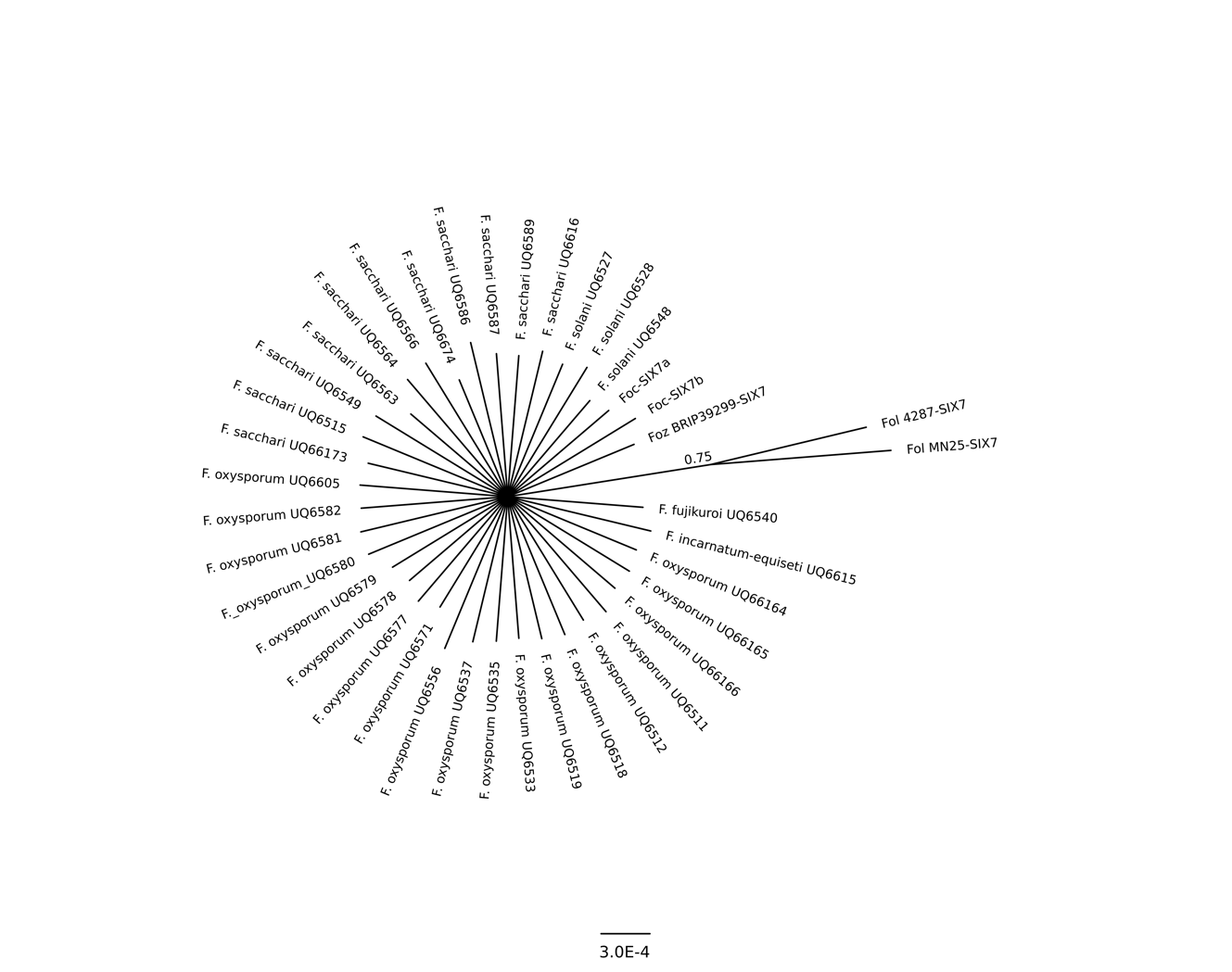

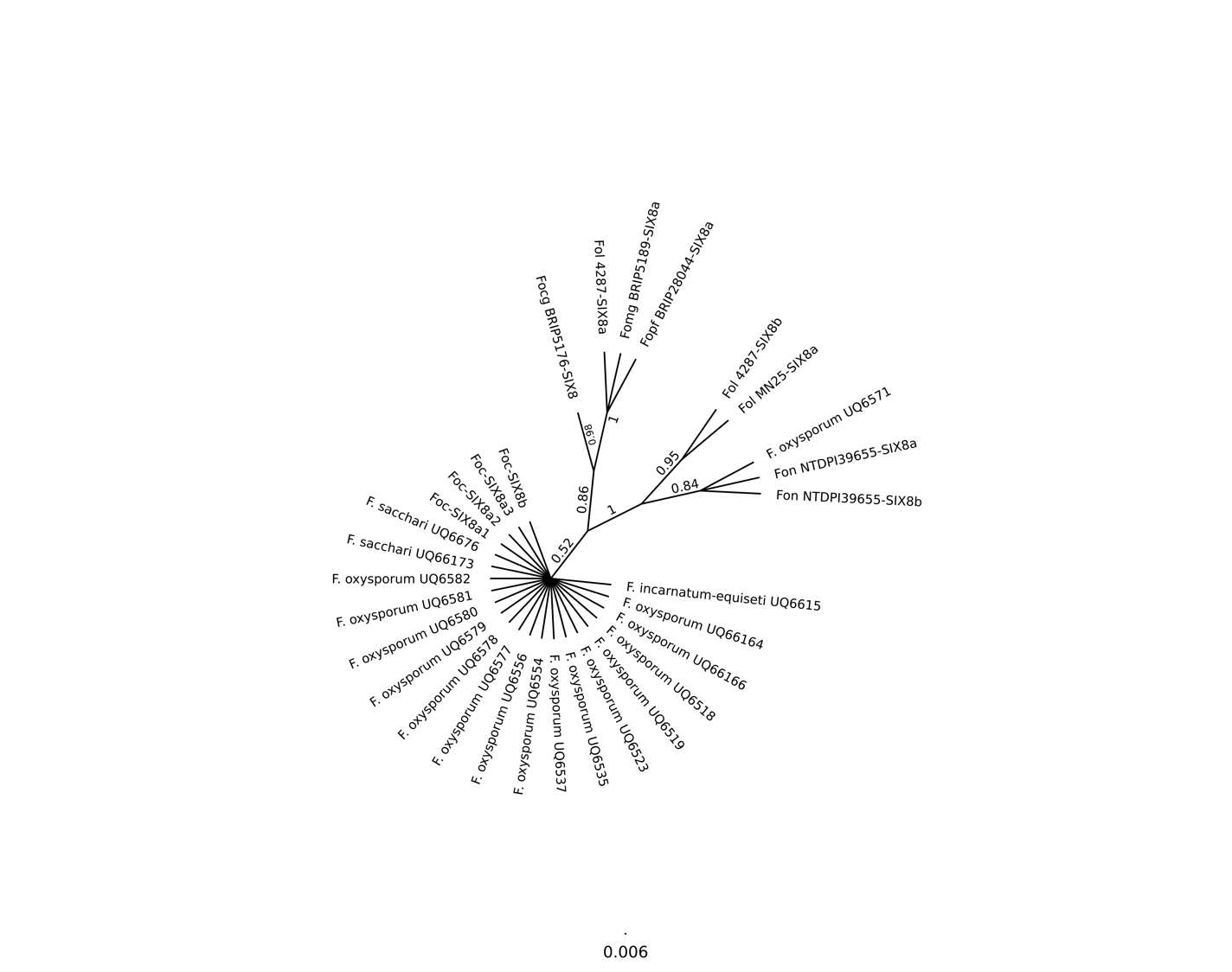


Zz

Zz

*SIX6*

*SIX7*

Zz

*SIX8*


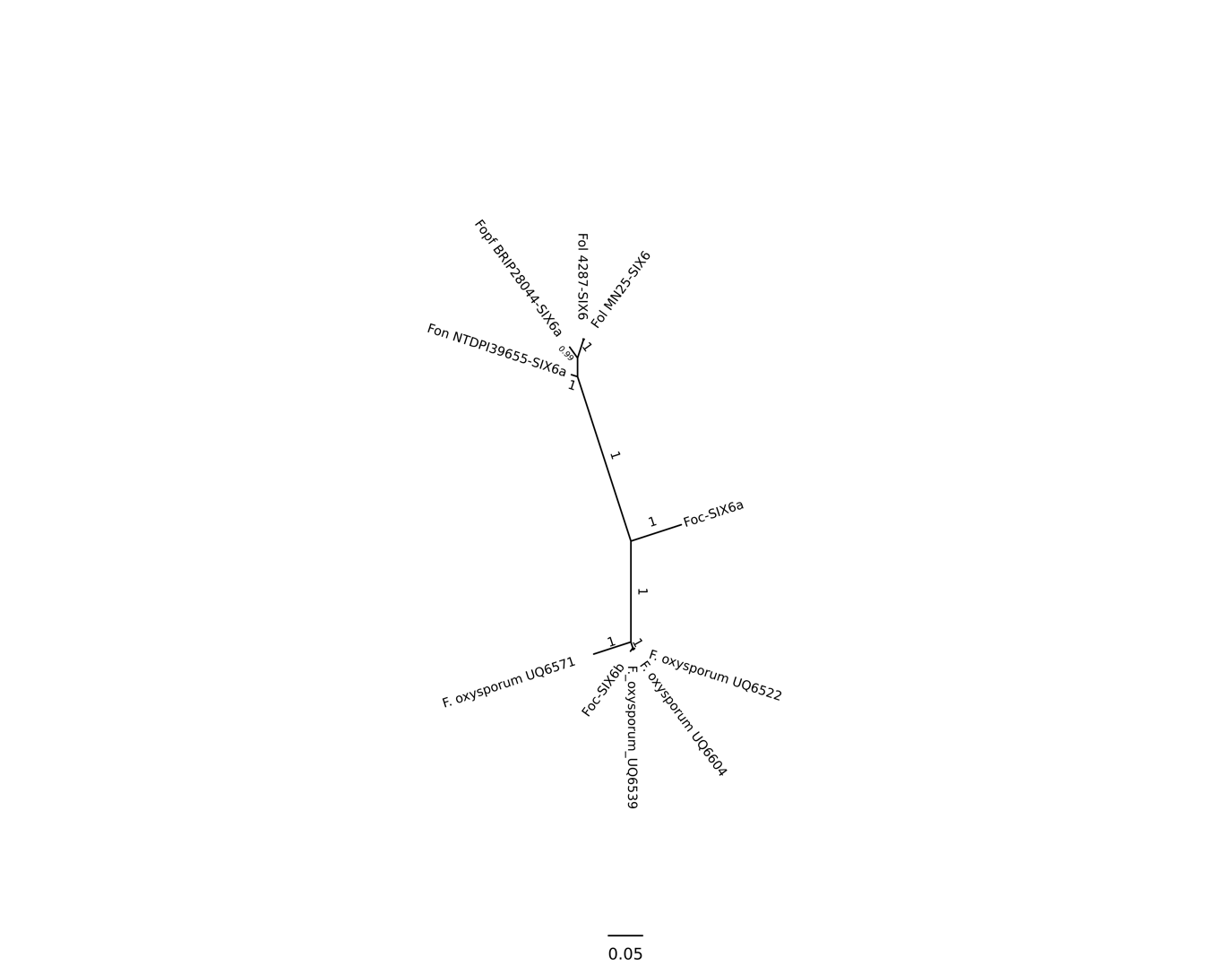


**Supplementary Figure 2** continued…


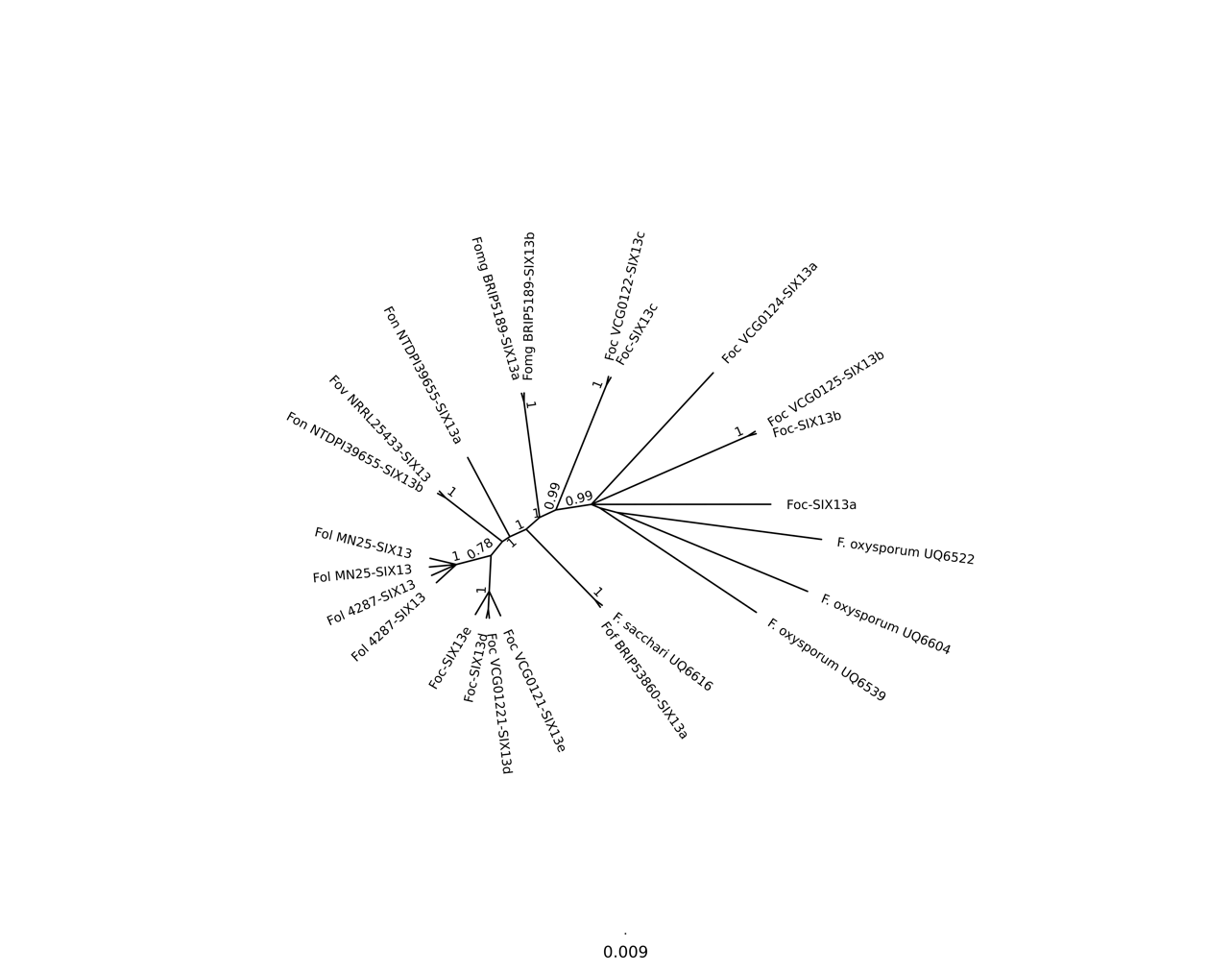

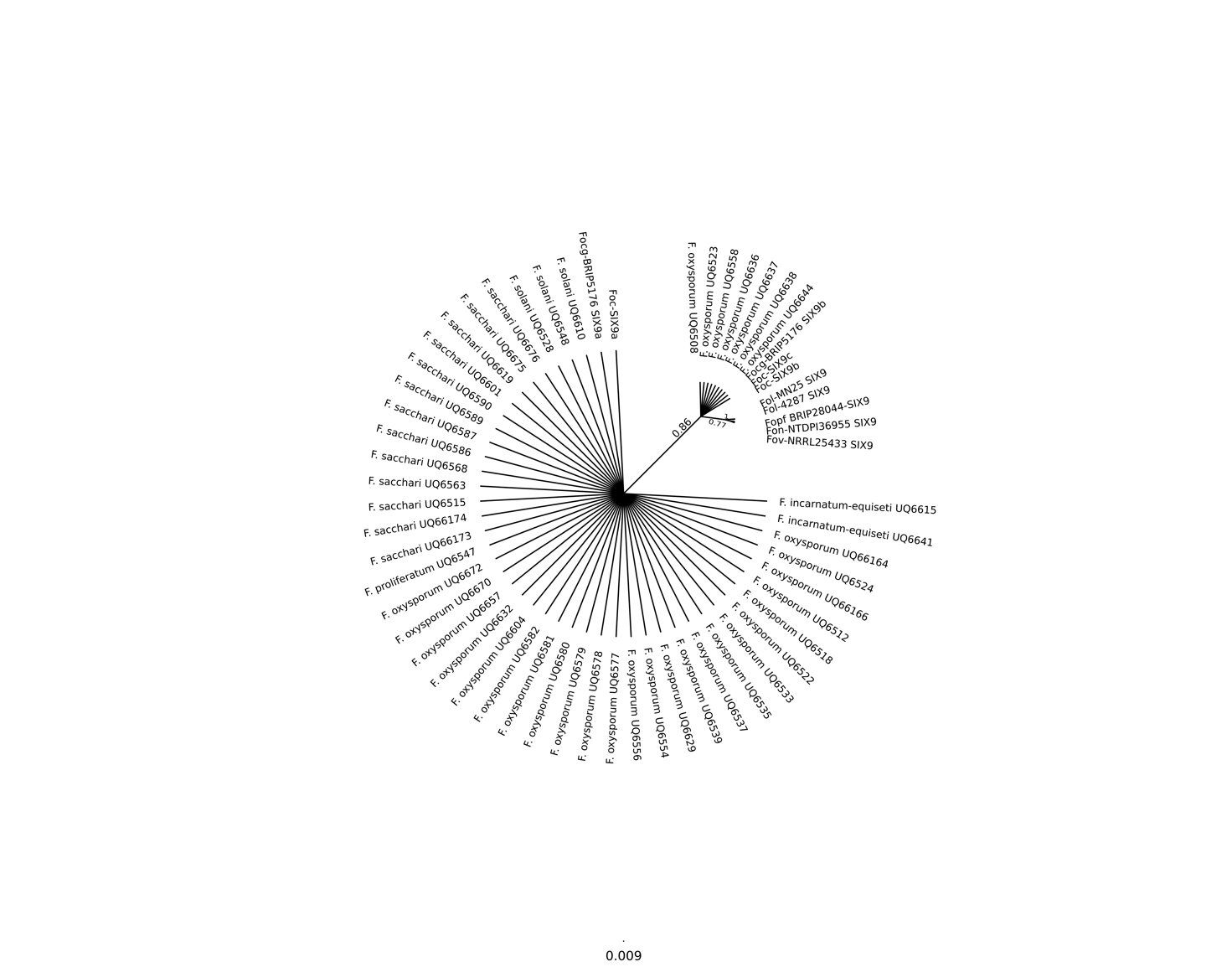


*SIX13*

*SIX12*

*SIX9*

Zz

Zz

Zz


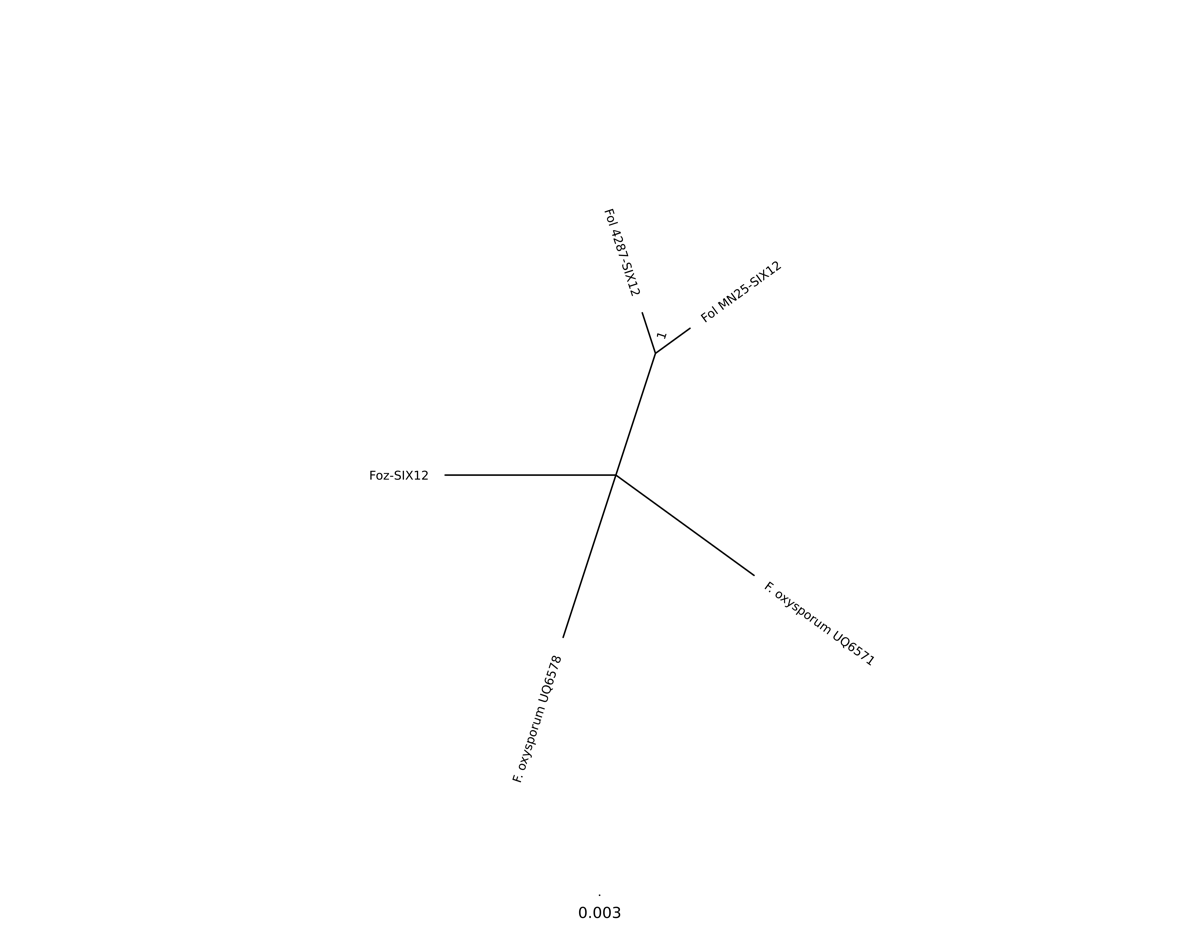


**Supplementary Figure 2** continued…

Zz


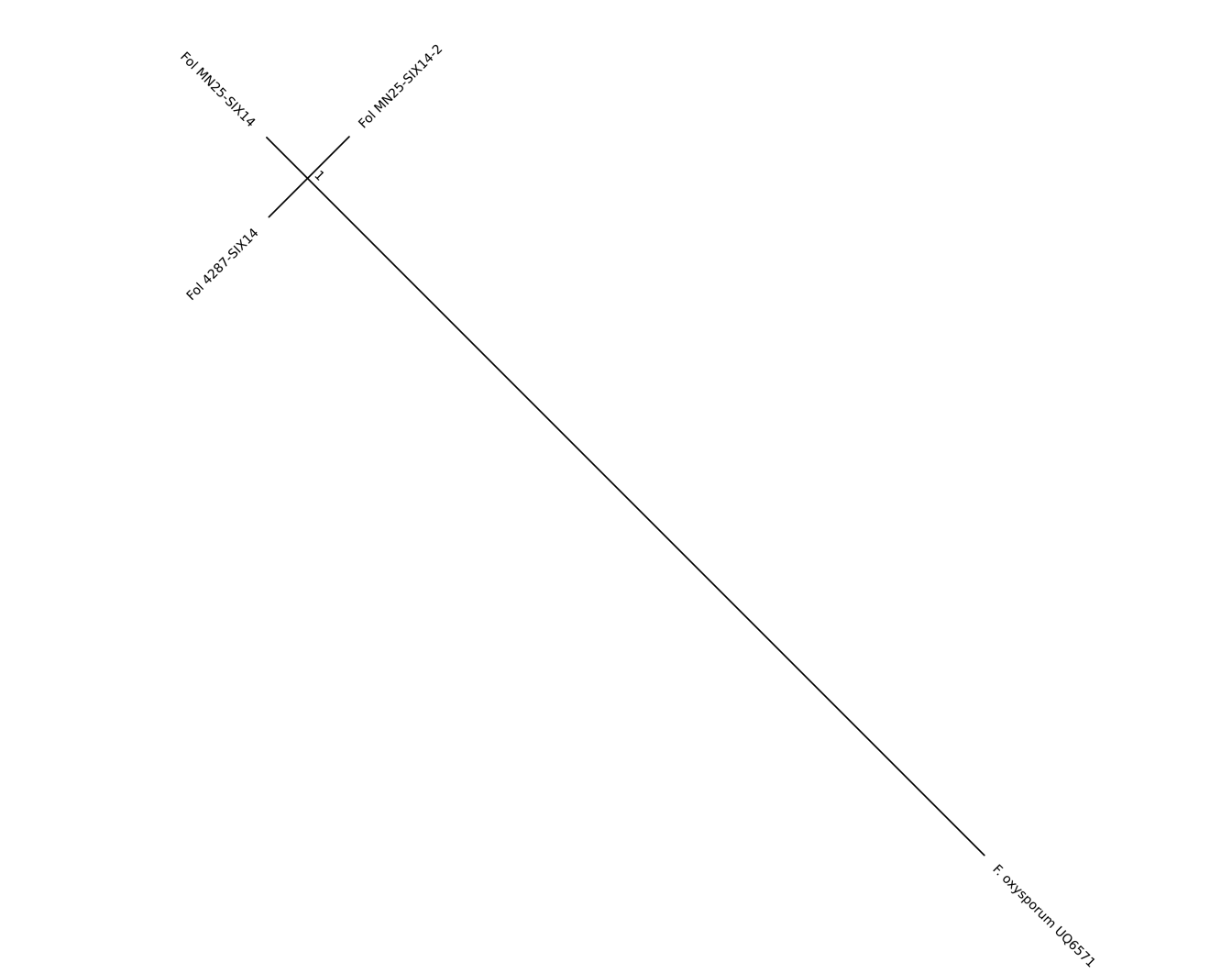


*SIX14*
